# Napabucasin, a novel STAT3 inhibitor suppresses proliferation, invasion and stemness of glioblastoma cells

**DOI:** 10.1186/s13046-019-1289-6

**Published:** 2019-07-05

**Authors:** Dongfeng Han, Tianfu Yu, Nan Dong, Bo Wang, Fei Sun, Dehua Jiang

**Affiliations:** 1 Department of Neurosurgery, Xuzhou Central Hospital, 199 Jie Fang Nan Road, Xuzhou, 221009 China; 2 Department of Neurosurgery, The Affiliated Drum Tower Hospital, School of Medicine, Nanjing University, Zhongshan Road 321, Nanjing, 210008 China; 3 Department of Neurosurgery, the First Affiliated Hospital of Nanjing Medical University, Guangzhou Road 300, Nanjing, 210029 China

**Keywords:** Glioma, STAT3, Inhibitor, Cancer stem cell

## Abstract

**Background:**

Glioblastoma (GBM) cells with stem cell-like properties are called glioma stem cells (GSCs). GSCs display highly treatment resistance and are responsible for tumor recurrence. Napabucasin (BBI608), a novel small molecule inhibitor of STAT3, has been identified to eliminate stemness-like tumor cells in some cancers. However, the influence of Napabucasin on GBM cells, especially on GSCs, is currently unclear. In this study, we explored the influence and underlying mechanisms of Napabucasin on GBM cells.

**Methods:**

STAT3 expression and its correlation with the glioma grade and patient survival were analyzed using CGGA and TCGA glioma databases. The influence of Napabucasin on proliferation, stemness, the cell cycle, apoptosis, and invasion of human GBM cell lines U87MG and LN229 was tested by CCK8, EdU incorporation, colony formation, Transwell invasion, and three-dimensional spheroid assays as well as flow cytometry, qPCR, and western blot analysis. The ability of Napabucasin to inhibit cell proliferation of U87MG tumor xenografts in mice was assessed using a live animal bioluminescence imaging system and immunohistochemistry.

**Results:**

Napabucasin suppressed the proliferation, colony formation, and invasion of U87MG and LN229 cells. Furthermore, Napabucasin induced cell cycle arrest and apoptosis. More importantly, Napabucasin treatment obviously inhibited expression of stemness-associated genes including STAT3 and suppressed the spheroid formation of glioma cells in vitro. Napabucasin also disrupted the NF-κB signaling pathway via downregulation of RelA (p65). Finally, glioma growth was effectively impaired by Napabucasin in nude mice bearing intracranial glioma xenografts.

**Conclusions:**

Napabucasin treatment may be a novel approach for the treatment of GBM, particularly GSCs.

## Background

Glioblastoma (GBM) is the most common and heterogeneous primary brain tumors in adults, accounting for more than 50% of glioma cases. It is also one of the most lethal cancers and challenging to treat [1]. Comprehensive standard therapies for GBM are maximal surgical resection, radiation, and chemotherapy using the alkylating drug temozolomide. Although many aggressive therapeutic methods are employed, the prognosis for GBM patients remains poor with median overall survival (OS) ranging from 14.6 to 16.8 months [2, 3]. Despite the further characterization of the distinct molecular alterations in GBMs by large-scale gene-expression studies, multiple clinical trials of novel therapies for GBM patients have failed to improve OS [4]. Glioma stem cells (GSCs) are defined by their ability for self-renewal, multilineage differentiation, and tumorigenicity meditated by various signaling pathways responsible for treatment resistance in GBMs [5]. Chemotherapy resistance is an intrinsic property of GSCs, which is acquired via multiple independent mechanisms including an increase of drug efflux pumps, an enhanced DNA repair capacity, and protection against reactive oxygen species [6]. Because GSCs contribute to glioma initiation, propagation, and recurrence, they are a crucial target of anti-GBM therapies.

Various molecular signaling pathways have been identified as prognostic markers or therapeutic targets for GBM [7]. Dysregulation of signal transducer and activator of transcription 3 (STAT3), a classic oncogenic transcription factor regulating the expression of a wide range of genes, has been reported in 50–90% of all human cancers including GBM [8]. Abundant evidence has highlights the essential roles of STAT3 in GSCs [9, 10]. Furthermore, STAT3-targeting agents to generate potent anti-glioma effects in the clinic remain to be further explored. Napabucasin (BBI608) is a newly developed small molecule inhibitor of STAT3, which has been shown to impair self-renewal and induce apoptosis in cancer stem cells of colorectal, pancreatic, non-small cell lung, gastric, and prostate cancers [11–15]. Importantly, Napabucasin is an orally administered agent. Clinical trials have been performed, many of which were in combination with numerous chemotherapeutic agents [16–18].

In this study, we found that Napabucasin effectively suppressed the proliferation, invasion, and sphere formation ability of GBM cells. It also arrested the cell cycle and induced apoptosis of GBM cells in vitro. Napabucasin decreased the expression of STAT3 and stemness-associated genes. Furthermore, Napabucasin displayed potent activity against tumor growth in an orthotopic nude mouse model of GBM. Taken together, Napabucasin treatment may be a novel approach to suppress GBM progression and improve its prognosis.

## Methods

### Ethics statement

This study was performed in accordance with the ethical standards according to the Declaration of Helsinki and national and international guidelines and was approved by The Research Ethics Committee of Nanjing Medical University.

### Cell culture and STAT3 inhibitor

Human GBM cell lines U87MG and LN229 used in this research were purchased from the Chinese Academy of Sciences Cell Bank (Shanghai, China), and were cultured in Dulbecco’s modified Eagle’s medium (DMEM, Hyclone, UT, USA) supplemented with 100 units of penicillin/mL, 100 ng of streptomycin/mL, and 10% fetal bovine serum (Gibco, MD, USA). These cells were incubated at 37 °C in a humidified atmosphere with 5% CO_2_. Cell lines were routinely tested for absence Mycoplasma. Napabucasin was purchased from MedChem Express (New Jersey, USA).

### Cell counting kit-8 assay

U87MG and LN229 were plated in 96-well plates (2000 cells/well) in triplicate and were incubated at 37 °C overnight. Subsequently, the cells were treated with Napabucasin (1, 5, and 10 μM) or vehicle control (0.1% DMSO) for 24, 48, and 72 h. Finally, the cell growth was measured by CCK-8 Cell Counting Kit (Dojindo, Japan) following the manufacturer’s protocols. Briefly, after one-hour incubation with CCK-8 at 37 °C, OD value (450 nm) was detected for calculating cell viability.

### 5-ethynyl-29-deoxyuridine (EdU) proliferation assay

To test the growth activity of U87MG and LN229 cells, Click-iT EdU Alexa Fluor 594 Imaging Kit (Thermo Fisher Scientific, Massachusetts, USA) was used according to the manufacturer’s protocol. After treated with 5 μM GSK343 or 0.1% DMSO for 48 h, the proportion of cells incorporated EdU was determined with fluorescence microscopy (Nikon, Tokyo, Japan).

### Colony formation assay

For the colony formation assay, cells were seeded in six-well plates (300 cells/well) and cultured for approximately 2 weeks until colony formation was observed. Colonies were fixed with methanol and stained with 1% crystal violet (Sigma, USA). The number of colonies was counted to determine colony-forming efficiency.

### Wound healing assay

U87MG and LN229 cells were seeded in 6-well plates and were cultured until reached 100% confluence. Next, a ten-microliter sterile pipette tip was used to create scratches across the cell monolayer and the photographs of scratched areas were taken. Then, cells were treated with 1 μM Napabucasin or vehicle control. One day later, a total of nine scratched areas were selected randomly in each well and were photographed under an inverted microscope. The cells protruding from the border of the scratches were counted.

### Transwell invasion assay

24-well plates using Transwell inserts (Corning, New York, USA) which were pre-coated with Matrigel (BD Biosciences, New Jersey, USA) were used to measure cell invasion. The upper chambers were added with 50,000 U87MG and LN229 cells which were treated with 1 μM Napabucasin or 0.1% DMSO and were dissolved in 200 μL serum-free media. In parallel, 900 μL DMEM media with 10% FBS was added to the lower chamber of each well. After incubation for 24 h at 37 °C, cells from the upper surface of the membrane were removed with a cotton swab and the penetrated cells were fixed with 4% methanol for 5 min and then stained with 0.1% crystal violet for 30 min. In each well, six fields of cells were captured and counted under an inverted microscope.

### Three-dimensional spheroid assays

U87MG and LN229 cells were seeded at a 2000 cells/ml density in 96-well ultra-low adherence plates (Corning, New York, USA). Over the course of 96 h these cells were induced to aggregate into a multicellular spheroid and then Matrigel mixed with 1 μM Napabucasin or 0.1% DMSO was added into wells. After 48 h, the motion of cells was confirmed as fully formed under light microscopy.

### Flow cytometry analysis

U87MG and LN229 cells were harvested and washed with phosphate-buffered saline (PBS). Then, 75% ethanol was used to fix cells at − 20 °C overnight. Next, DNA in cells was stained by Hank’s balanced salt solution including 50 mg/mL propidium iodide and 50 mg/mL RNaseA for 1 h at room temperature. Finally, the cell cycle of these cells was analyzed by Gallios flow cytometer (Beckman Countler).

### RNA extraction and quantitative real-time PCR

RNA was extracted from cells by TRIzol reagent (Invitrogen, Darmstadt, Germany). TaqMan real-time reverse transcription PCR was carried out for synthesizing cDNA by ABI StepOnePlus system (Applied Biosystems, Massachusetts, USA). Then, quantitative real-time PCR analysis was performed using ABI PRISM 7500 FAST Real-TIME PCR System. The relative expression was determined through the CT method and the results were normalized to GAPDH expression.

GAPDH, forward 5′-GCACCGTCAAGGCTGAGAAC-3′ and reverse 5′-TGGTGAAGACGCCAGTGGA-3′;

STAT3, forward 5′-CAGCAGCTTGACACACGGTA-3′ and reverse 5′-AAACACCAAAGTGGCATGTGA-3′;

β-catenin (CTNNB1), forward 5′-AAAGCGGCTGTTAGTCACTGG-3′ and reverse 5′-CGAGTCATTGCATACTGTCCAT-3′;

SOX2, forward 5′-GCCGAGTGGAAACTTTTGTCG-3′ and reverse 5′-GGCAGCGTGTACTTATCCTTCT-3′;

OCT4 (POU5F1), forward 5′-GTGTTCAGCCAAAAGACCATCT-3′ and reverse 5′-GGCCTGCATGAGGGTTTCT-3′;

NESTIN, forward 5′-CTGCTACCCTTGAGACACCTG-3′ and reverse 5′-GGGCTCTGATCTCTGCATCTAC-3′;

### Western blot analysis

RIPA lysis buffer (KeyGEN, Jiangsu, China) was used to extract proteins from cells. Equal amounts of protein were separated by SDS-PAGE, followed by electro-transfer onto poly-vinylidene difluoride membranes (Thermo Fisher Scientific, Massachusetts, USA). Then, membranes were blocked for 2 h with 5% nonfat milk and then incubated at room temperature with the primary antibody. After incubating with secondary antibody for 2 h, membranes were developed using an enhanced chemiluminescence detection system (GE Healthcare, Chicago, USA). All the antibodies were obtained from Cell Signaling Technology (Massachusetts, USA).

### Fluorescent staining

Cells fixed by 4% formalin were incubated with rabbit monoclonal anti-cleaved caspase-3 overnight at 4 °C and then with Alexa 488-labeled anti-rabbit IgG antibody (Thermo Fisher Scientific, Massachusetts, USA) 2 h at room temperature. After treated with DAPI (Beyotime Biotechnology, Jiangsu, China) for 10 min, cells were examined with Zeiss Axiophot photomicroscope (Carl Zeiss AG, Jena, Germany).

### In vivo experiments, IHC, and H&E staining

Animal experiments were approved by the Animal Management Rule of the Chinese Ministry of Health (documentation 55, 2001) and were in accordance with the approved guidelines and the experimental protocol of Nanjing Medical University. 6-week-old female nude mice purchased from Cancer Institute of the Chinese Academy of Medical Science were implanted with 1 × 10^6^ luciferase-expressing U87MG cells per mouse (Napabucasin-treated group, *N* = 6; DMSO-treated group, N = 6). After 7 days, nude mice received either vehicle (10 μL DMSO in 200 μL PBS) or Napabucasin (40 mg/kg in 200 μL PBS) intraperitoneally every other day. Tumor growth was assessed weekly by live animal bioluminescence imaging system.

### Statistical analysis

To analyze differences in each two-group comparison, t-test was employed while one-way ANOVA was performed to determine the difference among at least three groups. Kaplan–Meier analysis was used to assess the survival rate of mice. *P* < 0.05 was considered statistically significant.

## Results

### Napabucasin inhibits glioma cell proliferation and represses the expression of STAT3

**Fig. 1:**
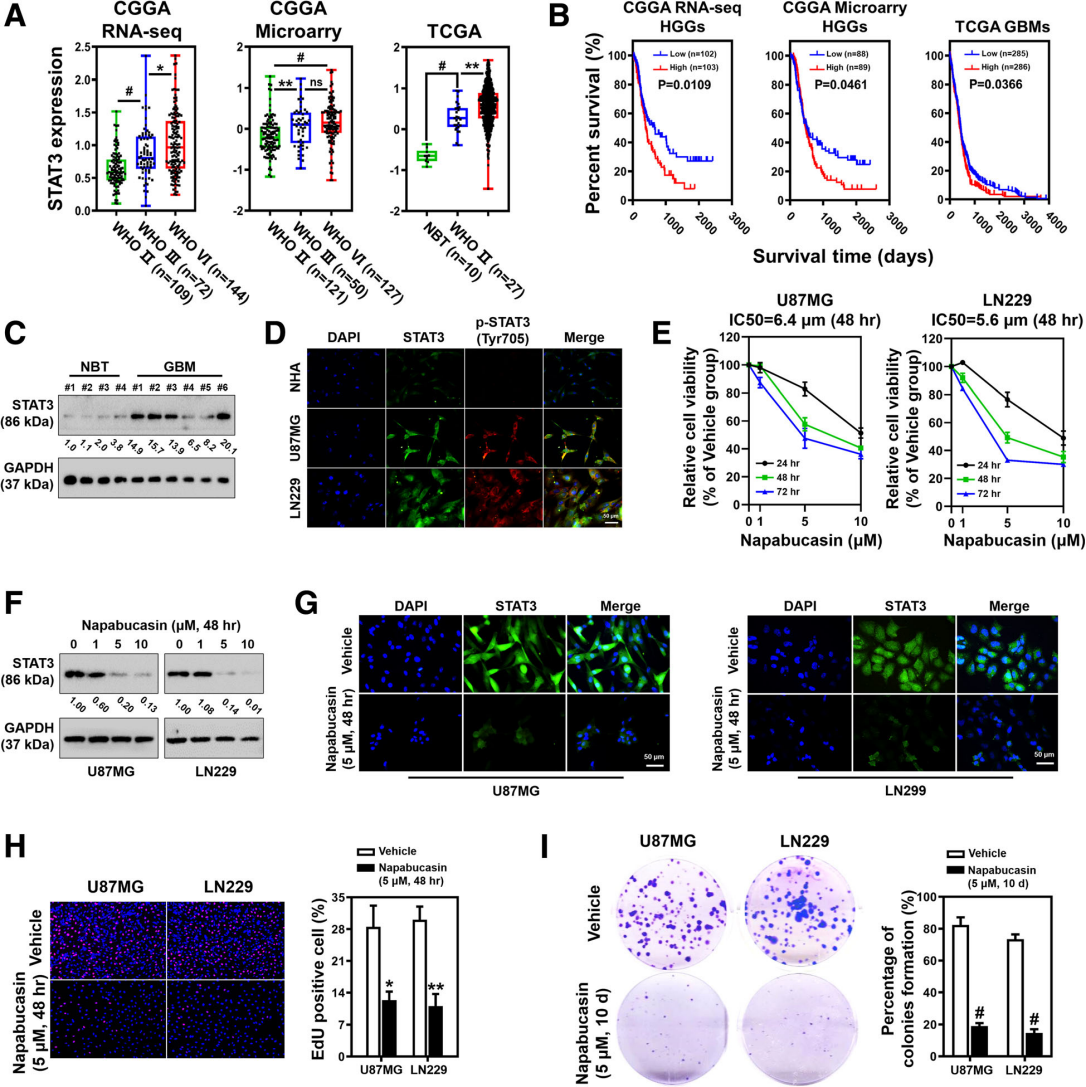
Napabucasin inhibits GBM cell proliferation and STAT3 expression. **a** Correlation of STAT3 mRNA expression and the glioma grade in CGGA and TCGA databases. **b** Kaplan-Meier (KM) survival curves comparing glioma patients stratified by expression of STAT3. **c** Western blot analysis of STAT3 expression in four normal brain tissues (NBTs) and six GBM tissues. **d** Expression and localization of total STAT3 and p-STAT3 detected by immunofluorescence (IF) in normal human astrocytes (NHAs), U87MG and LN229 cells (scale bar: 50 μm). **e** Proliferation of glioma cells treated with Napabucasin and detected by CCK-8 assays. Half maximal inhibitory concentrations (IC_50_) of Napabucasin in U87MG and LN229 cells were 6.4 and 5.6 μM respectively. **f** After treatment with the indicated doses of Napabucasin (0, 1, 5, and 10 μM) for 48 h, protein levels of STAT3 in U87MG and LN229 cells were evaluated by western blotting. **g** IF was used to estimate the expression of STAT3 in U87MG and LN229 cells after treatment with Napabucasin (scale bar: 50 μm). **h** EdU incorporation assays showed that exposure to 5 μM Napabucasin for 48 h impaired DNA synthesis in U87MG and LN229 cells. **i** Clonogenic assays were performed to examine the survival and proliferation of glioma cells treated with the vehicle or Napabucasin. (*p < 0.05, **p < 0.01, #*p* < 0.001)

Previous studies have shown that STAT3 is a therapeutic target in a broad spectrum of cancers. To further confirm the therapeutic value of targeting STAT3 in glioma, two independent glioma cohorts from the CGGA (Chinese Glioma Genome Atlas) mRNA profile databases and TCGA (The Cancer Genome Atlas) glioma database were used to investigate STAT3 in clinical samples. Figure 1a shows that the expression of STAT3 was increased in high grade gliomas [HGGs; World Health Organization (WHO) III and IV] compared with low-grade gliomas (LGGs, WHO II) (*P* < 0.001) in two CGGA databases. In the TCGA glioma dataset, STAT3 expression was also upregulated in GBMs (WHO IV) compared with LGGs (*P* < 0.001) and normal brain tissues (NBTs) (*P* < 0.0001). Additionally, patients with high STAT3 expression had reduced survival times compared with patients with low STAT3 expression (Fig. 1b). Next, we measured the expression of STAT3 in four NBT samples and six GBM samples. Western blot analysis showed that STAT3 protein levels were dramatically increased in GBM samples (Fig. 1c). Then, the basal expression level and phosphorylation (Tyr705) of STAT3 were examined by immunofluorescence (IF). Compared with normal human astrocytes (NHAs), the expression and phosphorylation levels of STAT3 in U87MG and LN229 cells were elevated significantly (Fig. 1d). To explore the anti-glioma activity of Napabucasin in vitro, we first tested its effect on glioma cell proliferation. The viability of U87MG and LN229 cells was evaluated by CCK8 assays after treatment with various concentrations of Napabucasin (1, 5, and 10 μM) or the vehicle control (0.1% DMSO) for 24, 48, and 72 h. As shown in Fig. 1e, Napabucasin significantly impaired the proliferation of U87MG and LN229 glioma cells in a time- and dose-dependent manner. Furthermore, the results revealed that 6.4 and 5.6 μM were the half maximal inhibitory concentrations (IC50) in U87MG and LN229 cells, respectively. Next, glioma cells were treated with Napabucasin or the vehicle for 48 h and then subjected to western blotting. We found that the expression of STAT3 was significantly decreased compared with the control group (Fig. 1f). Additionally, IF confirmed that Napabucasin remarkably reduced the expression of STAT3 (Fig. 1g). DNA synthesis in Napabucasin- and vehicle-treated GBM cells was assessed by the Click-iT EdU Imaging Kit. Treatment with 5 μM Napabucasin resulted in a marked decrease in the fraction of EdU-positive cells compared with untreated cells at 48 h after treatment in U87MG (27.9 ± 3.2% vs. 12.3 ± 1.7%, *p* = 0.011) and LN229 cells (30.1 ± 2.6% vs. 11.7 ± 2.3%, *p* = 0.0005) (Fig. 1h). Compared with control groups, the percentages of colony-forming U87MG cells (82.4 ± 4.3% vs. 19.0 ± 1.7%, *p* < 0.0001) and LN229 cells (73.4 ± 2.7% vs. 14.7 ± 2.1%, *p* < 0.0001) were also reduced after Napabucasin treatment (Fig. 1i). Collectively, Napabucasin effectively reduced cell proliferation and the protein level of STAT3 in vitro.

### Napabucasin arrests the cell cycle in GBM cells and induces apoptosis

**Fig. 2:**
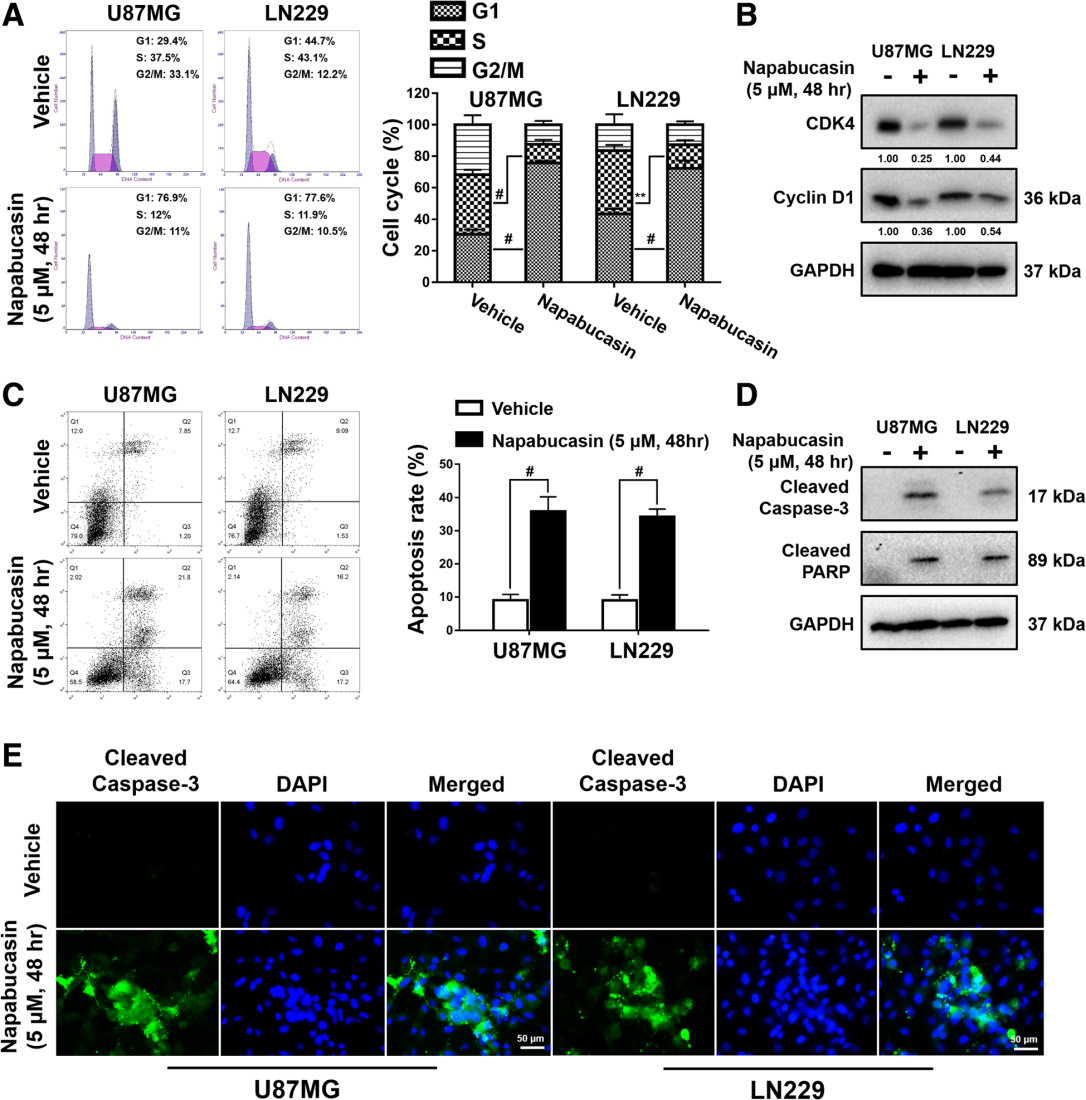
Napabucasin arrests the GBM cell cycle and induces apoptosis. **a** Cell cycle distribution of glioma cells analyzed by flow cytometry. **b** U87MG and LN229 cells were treated with the vehicle or 5 μM Napabucasin for 48 h. Then, cell lysates were analyzed by western blotting. **c** Annexin V-APC/7-AAD staining was performed for apoptosis detection by flow cytometry. **d** Western blot analysis of cleaved caspase-3 and cleaved PARP levels in glioma cells. **e** Immunofluorescence staining of glioma cells treated with the vehicle or Napabucasin. Nuclei were stained with DAPI (blue). Scale bar = 50 mm. (*p < 0.05, **p < 0.01, #*p* < 0.001)

We assumed that Napabucasin inhibited proliferation of U87MG and LN229 glioma cells through cell cycle arrest and/or induction of apoptosis. Flow cytometric analysis was used to clarify whether Napabucasin influenced the cell cycle of glioma cells. As shown in Fig. 2a, Napabucasin treatment (5 μM, 48 h) led to accumulation of cells in G0/G1 phase and a reduction of cells in S phase in the U87MG cell line (G0/G1 phase: 30.5 ± 2.5% vs. 76.7 ± 1.8%, p < 0.0001; S phase: 37.8 ± 2.5% vs. 11.9 ± 2.3%, *p* = 0.0004) and LN229 cell line (G0/G1 phase: 43.4 ± 2.6% vs. 72.3 ± 4.1%, *p* = 0.001; S phase: 40.1 ± 3.0% vs. 15.0 ± 2.4%, *p* = 0.0007) compared with untreated cells. Cyclin dependent kinase 4 (CDK4) is a key driver protein of the cell cycle and cooperates with cyclin D1 to promote G1/S transition. In conjunction with flow cytometric analysis of the cell cycle, western blotting demonstrated that Napabucasin treatment (5 μM, 48 h) reduced the protein levels of CDK4 and cyclin D1 (Fig. 2b). Next, the apoptotic ratio detected by the Annexin V-APC/7-AAD kit was elevated in U87MG cells (9.0 ± 1.4% vs. 35.9 ± 3.6%, *p* = 0.0006) and LN229 cells (9.0 ± 1.5% vs. 34.2 ± 1.9%, *p* = 0.0001) following exposure to 5 μM Napabucasin for 48 h (Fig. 2c). Further analysis showed that the expression of cleaved caspase-3 and cleaved PARP was strikingly enhanced in Napabucasin-treated GBM cells (Fig. 2d). Representative images of IF staining showed that cleaved caspase-3 was widely distributed in GBM cells treated with Napabucasin (Fig. 2e). Overall, these results indicated that Napabucasin effectively blocked G1/S transition and induced apoptosis in GBM cells.

### Napabucasin represses the migration and invasion of GBM cells

**Fig. 3:**
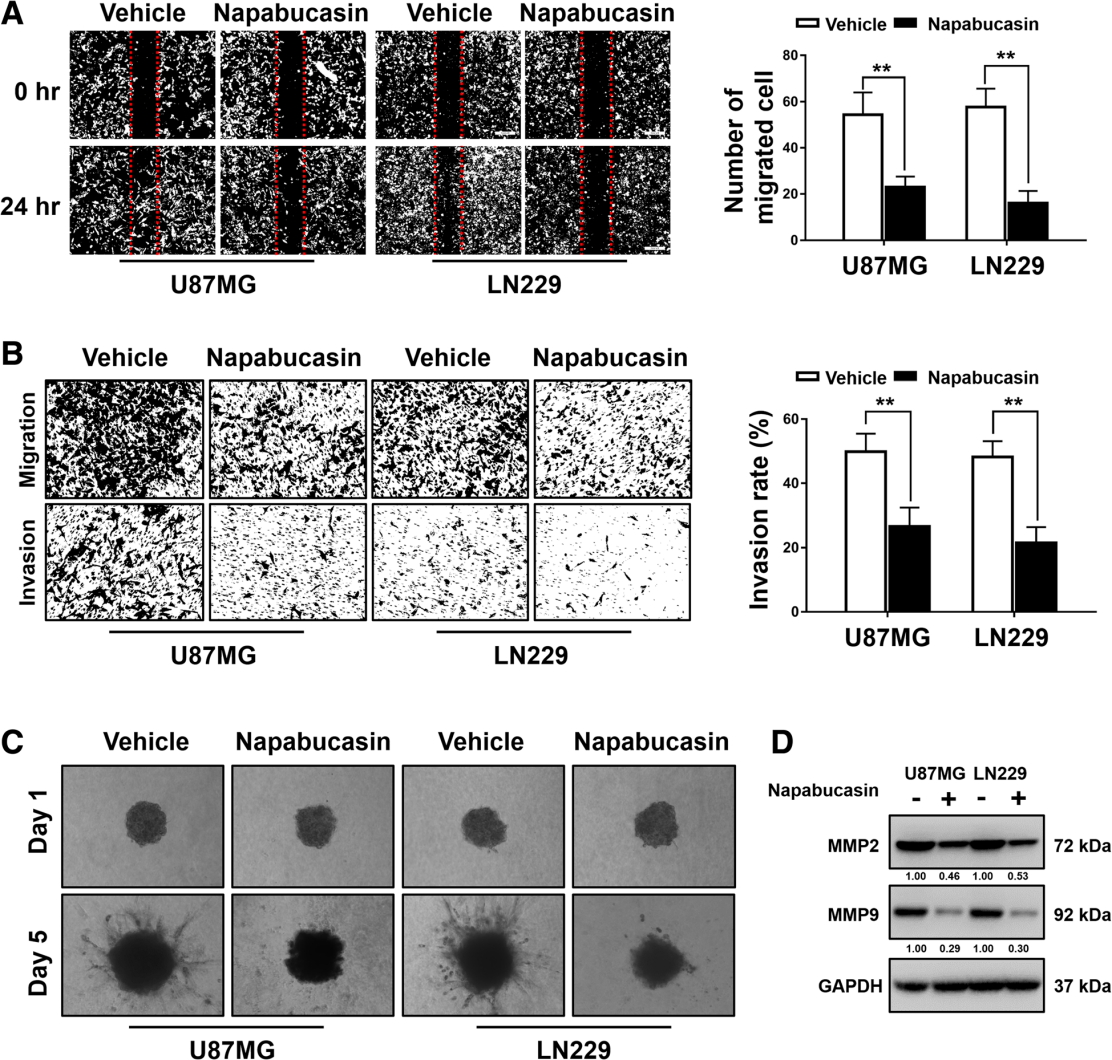
Napabucasin represses the migration and invasion of GBM cells. **a** Representative images of the wound healing assay showing that Napabucasin treatment (1 μM, 24 h) inhibited glioma cell migration. **b** Transwell assays were performed to evaluate the invasive abilities of glioma cells treated with the vehicle or Napabucasin (1 μM, 24 h). Quantification was carried out by counting the number of cells in the ‘Migration’ panel (inserts without Matrigel represent migrated cells) and ‘Invasion’ panel (inserts with Matrigel represent invasive cells). The invasive ability of glioma cells is indicated by the invasion index (‘Invasion’ panel/ ‘Migration’ panel× 100%). **c** Three-dimensional spheroid cell invasion assay of glioma cells treated with the vehicle or Napabucasin (1 μM). Representative images (× 100) were captured at days 1 and days 5. **d** Western blot analysis showed that the levels of MMP2 and MMP9 were significantly reduced in the Napabucasin (1 μM, 24 h) treated group. (*p < 0.05, **p < 0.01, #*p* < 0.001)

According to the results shown in Fig. 1e, treatment with 1 μM Napabucasin for 24 h did not influence the viability of U87MG and LN229 cells. Thus, to avoid a reduction in cell number, we chose 1 μM Napabucasin to investigate cell migration and invasion. Napabucasin-treated U87MG and LN229 cell groups had compromised wound closure compared with the vehicle control group (U87MG: 54 ± 8 vs. 23 ± 4, *P* = 0.007; LN229: 58 ± 7 vs. 58 ± 7, *P* = 0.001) (Fig. 3a). Next, we carried out Transwell assays and found that fewer U87MG and LN229 cells invaded through the insert membranes after Napabucasin exposure (U87MG: 49.8 ± 4.6% vs. 26.5 ± 4.8%, *P* = 0.008; LN229: 48.2 ± 4.0% vs. 21.5 ± 3.9%, *P* = 0.002) (Fig. 3b). Moreover, the invasive ability of glioma cells treated with Napabucasin was investigated in a 3D environment. Napabucasin was applied to a spheroid suspension after collection and the medium of the 3D culture to facilitate adequate drug exposure to the cells. The resulting data were consistent with previous results (Fig. 3c). Finally, western blotting showed that the expression of MMP2 and MMP9 was decreased after Napabucasin treatment (Fig. 3d). These results showed that Napabucasin impaired the migration and invasion of GBM cells.

### Napabucasin impairs the stemness of GBM cells

**Fig. 4:**
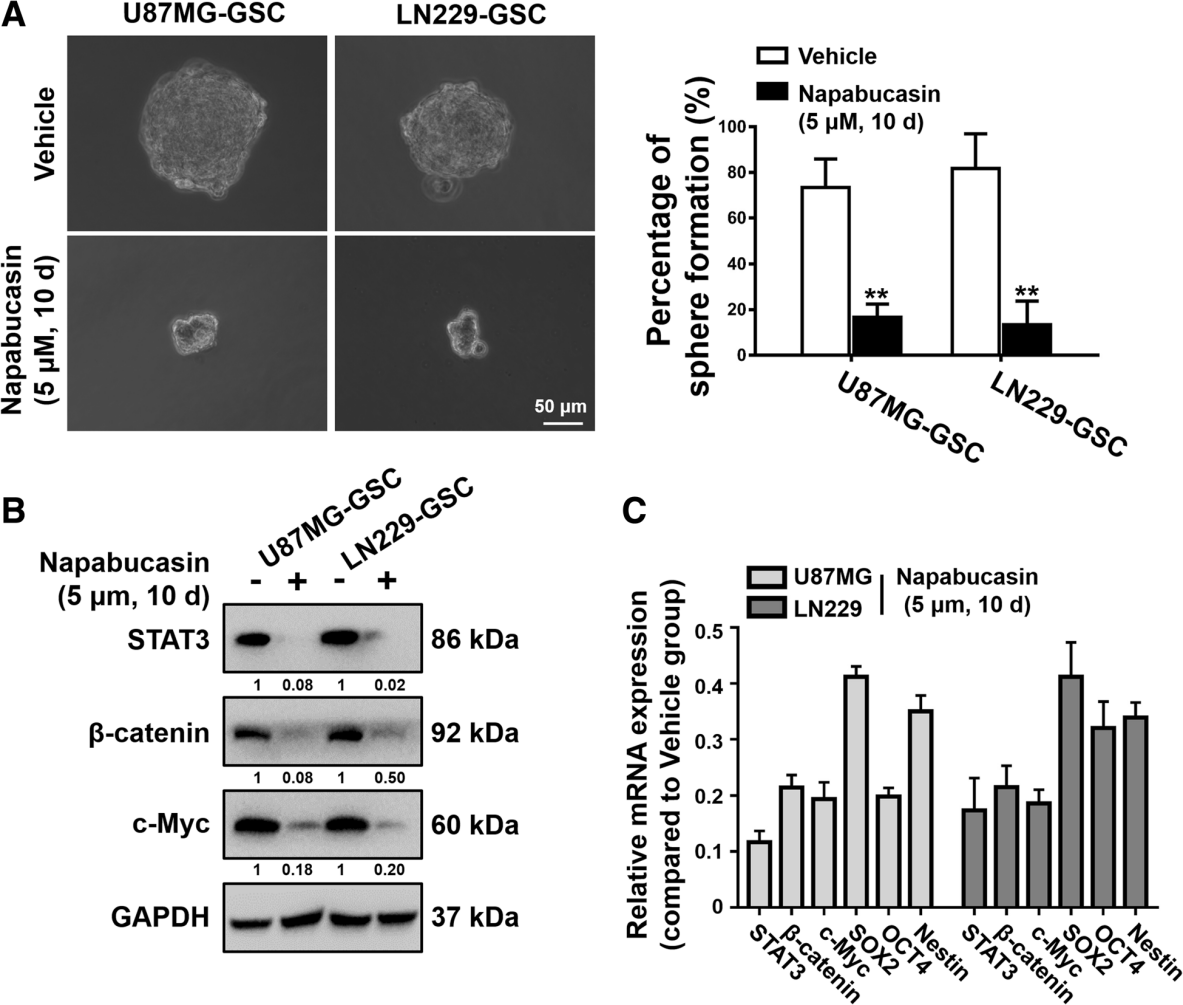
Napabucasin impairs the stemness of GBM cells. **a** GSCs from U87MG and LN229 cells were grown in serum-free DMEM/F12 medium with or without Napabucasin for 10 days. Sphere morphology was photographed under a light microscope and spheroids were counted. **b** GSCs under the indicated conditions were harvested and subjected to western blotting. **c** Transcriptional levels of stemness-associated markers (STAT3, β-catenin, c-Myc, Sox2, OCT4, and Nestin) in GSCs were determined by qPCR. (*p < 0.05, **p < 0.01, #*p* < 0.001)

To determine the effect of Napabucasin on glioma stem cells (GSCs), we examined the self-renewal ability of cells enriched by stem cell culture medium. Tumor spheres (U87MG-GSCs and LN229-GSCs) were dissociated into single cells and allowed to form spheres in cancer stem cell medium with or without Napabucasin (5 μM). After 10 days of incubation, the spheres were photographed, counted, and subjected to qPCR and western blotting. Treatment with Napabucasin restrained sphere formation of GSCs, suggesting that Napabucasin inhibits self-renewal of GSCs (U87MG-GSCs: 73.3 ± 10.3% vs. 16.7 ± 4.7%, *P* = 0.002; LN229-GSCs: 81.7 ± 12.5% vs. 13.3 ± 8.5%, *P* = 0.003) (Fig. 4a). Next, we investigated the mRNA and protein levels of several genes involved in glioma cell stemness. Napabucasin treatment decreased the expression of STAT3, β- catenin, and c-Myc in U87MG-GSCs and LN229-GSCs (Fig. 4b). Further analysis also showed a significant decrease in mRNA levels of STAT3 (11.7 ± 1.6% and 17.4 ± 4.7%), β-catenin (21.4 ± 1.9% and 21.5 ± 3.1%), c-Myc (19.4 ± 2.5% and 18.6 ± 2.0%), SOX2 (41.2 ± 1.5% and 40.5 ± 5.0%), OCT4 (19.8 ± 1.3% and 25.0 ± 2.1%), and Nestin (35.0 ± 2.3% and 33.9 ± 2.2%) in GSCs treated with Napabucasin (Fig. 4c). Therefore, Napabucasin blocked survival and self-renewal of glioma stem cells by downregulating the expression of STAT3 and other stemness markers.

### Napabucasin downregulates the expression and phosphorylation of RelA

**Fig. 5:**
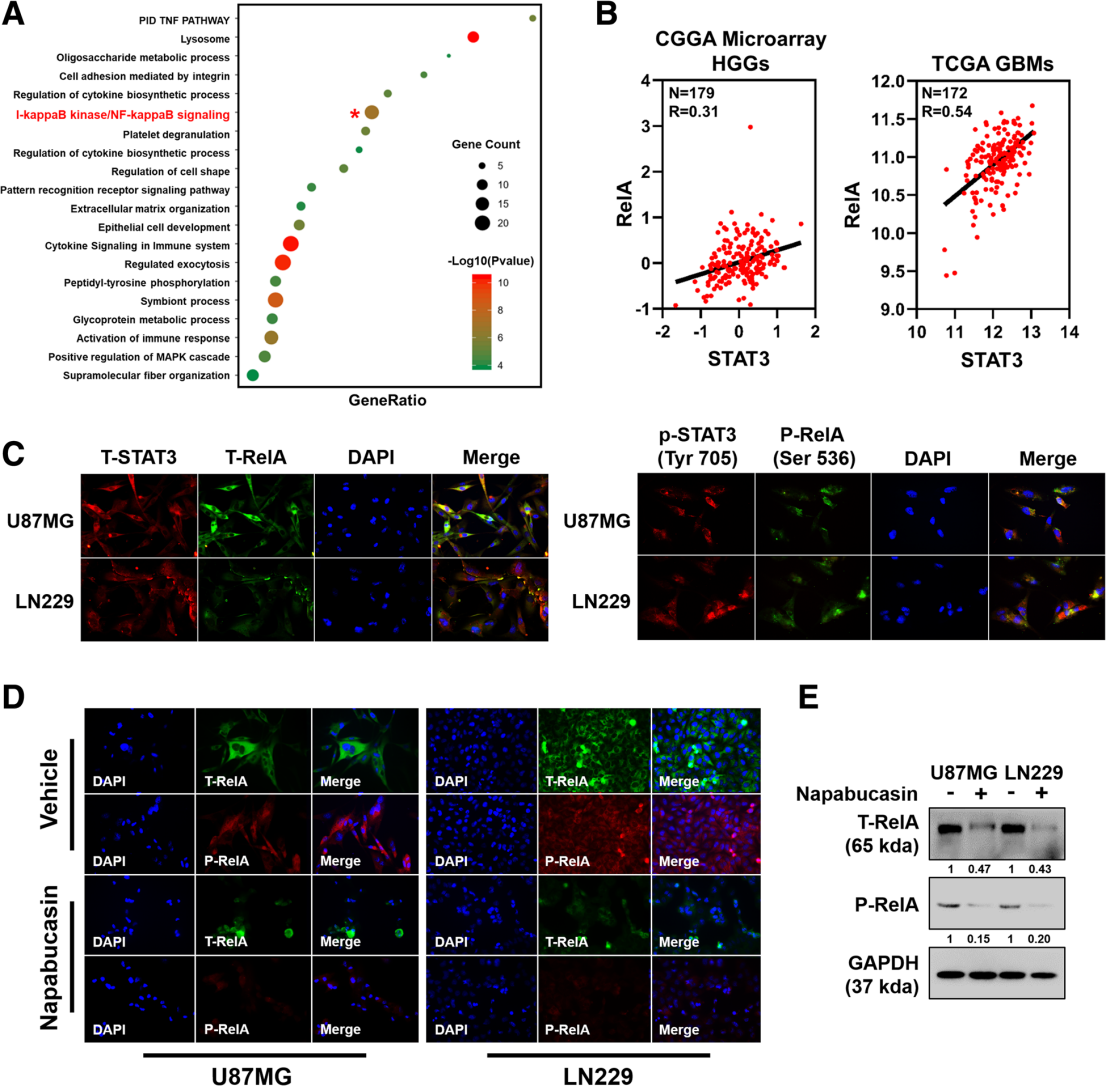
Napabucasin downregulates RelA and reduces constitutive p-RelA in GBM cells. **a** Bubble chart of upregulated genes analyzed by KEGG/GO. The top 20 enrichment pathways are displayed on the Y-axis. The bubble size represents the number of genes, and the bubble color represents the -log(*p*-value). **b** Correlations between STAT3 and RelA were analyzed in CGGA and TCGA databases. **c** Immunofluorescence (IF) localization of STAT3 (p-STAT3) and RelA (p-RelA) in U87MG and LN229 cells. Localization of STAT3 (p-STAT3) and RelA (p-RelA) is shown by red and green fluorescence, respectively. Colocalization of STAT3 (p-STAT3) and RelA (p-RelA) is shown by yellow fluorescence. Nuclei were stained with DAPI (blue). Scale bar = 50 μm. **d** IF of the expression and location of total RelA (t-RelA) and phospho-Ser536-RelA (p-RelA) in GBM cells after treatment with 5 μM Napabucasin for 48 h. Nuclei were stained with DAPI (blue). Scale bar = 50 μm. **e** U87MG and LN229 cells were treated with Napabucasin (5 μM) or DMSO (0.1%) for 48 h. Then, cell lysates were analyzed by western blotting using antibodies against t-RelA and p-RelA. (*p < 0.05, **p < 0.01, #*p* < 0.001)

To gain further insight into the influence of Napabucasin-induced STAT3 downregulation, Pearson correlation analysis was carried out to identify target genes that were positively associated with STAT3 in the CGGA and TCGA glioma databases. The selected genes were overlapped and analyzed using Metascape, a web-based portal providing comprehensive gene annotation and enrichment analysis including GO processes, KEGG pathways, and the Reactome gene set [19]. Except for the response to cytokines and growth factors, the STAT3 signaling pathway is closely coordinated with other signaling pathways. As shown in Fig. 5a, positively correlated genes were strongly enriched in the NF-κB signaling pathway. Furthermore, we found a significant positive correlation between STAT3 and RelA (p65), a core subunit of the NF-κB transcription factor family, in CGGA and TCGA databases (*r* = 0.31, *p* < 0.0001 and *r* = 0.54, *p* < 0.0001 respectively, Pearson correlation analysis) (Fig. 5b). STAT3 and NF-κB signaling pathways intricately cooperate in many pathological processes. However, it remains unclear whether downregulation of STAT3 induced by Napabucasin influences over-active NF-κB signaling in GBM cells. IF confirmed that RelA was colocalized with STAT3 in both phosphorylated and non-phosphorylated states (Fig. 5c). We next explored the influence of Napabucasin on the NF-κB pathway. Napabucasin treatment (5 μM, 48 h) successfully impaired activation of the NF-κB pathway as characterized by significantly decreased total and phospho-RelA fluorescence intensities (Fig. 5d). This observation was confirmed by a western blot assay, demonstrating that treatment with Napabucasin reduced the protein levels of total and phospho-RelA (Fig. 5e). These data indicated that Napabucasin effectively restrained the hyperactivity of the NF-κB pathway in U87MG and LN229 cells.

### Napabucasin suppressed tumor growth in an orthotopic glioma model

**Fig. 6:**
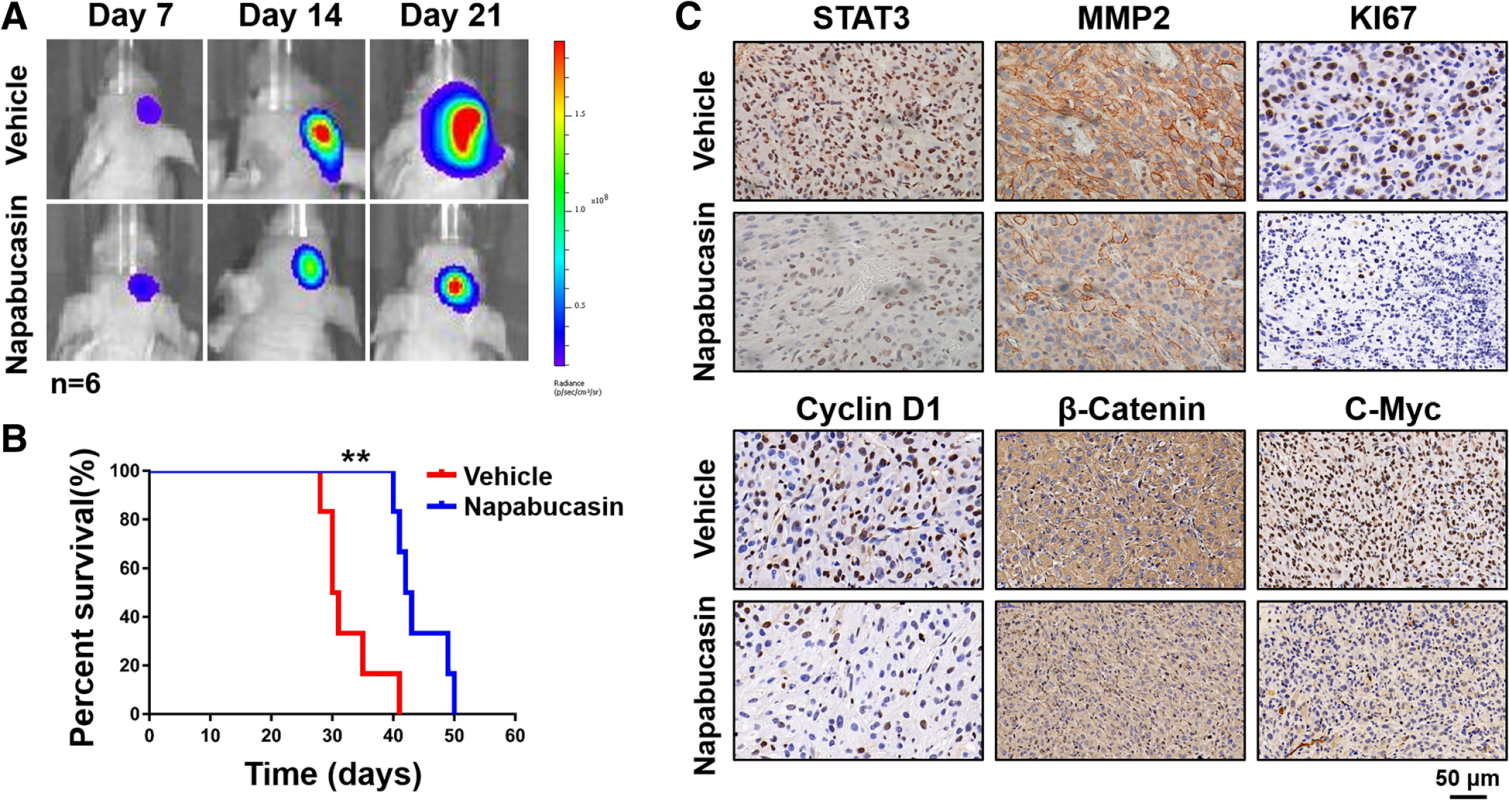
Napabucasin suppresses tumor growth in an orthotopic glioma model. **a** Changes in luciferase signals were detected at days 7, 14, and 21 after implantation of U87MG cells in situ (*n* = 6). The bioluminescent signal in the Napabucasin group was significantly lower than that in the vehicle group. **b** Survival differences between vehicle and Napabucasin groups shown by survival curves. The log-rank test was used to evaluate statistical differences. **c** Immunohistochemistry was used to assess the expression of STAT3, MMP2, Ki-67, cyclin D1, β-catenin, and c-Myc in orthotopic xenografted tumors after vehicle or Napabucasin treatments. (*p < 0.05, **p < 0.01, #*p* < 0.001)

Recent studies have revealed that Napabucasin significantly delays tumor growth and extends survival in mouse xenograft models of liver, pancreatic, and prostate cancers. Furthermore, clinical trials of Napabucasin are recruiting patients with colorectal cancer, pancreatic ductal carcinoma, and non-small cell lung cancers. However, the effect of Napabucasin on intracranial tumors is unknown. Therefore, nude mice were intracranially xeno-transplanted with luciferase-expressing U87MG glioma cells to establish an orthotopic glioma model. Next, glioma-carrying nude mice were treated with intraperitoneal injections of Napabucasin (40 mg/kg) or diluted DMSO (10 μL in 200 μL PBS) every other day and then monitored by the live animal bioluminescence imaging system every week. Figure 6a shows that gliomas in nude mice treated with Napabucasin exhibited significantly slower proliferation. Compared with the vehicle group, the Napabucasin-treated group had a prolonged survival period (30.5 days vs. 42.5 days, *p* = 0.003) (Fig. 6b). Consistent with the in vitro results, immunohistochemical analyses demonstrated that the expression levels of STAT3, MMP2, Ki-67, cyclin D1, β-catenin, and c-Myc were downregulated in the Napabucasin-treated group (Fig. 6c). Collectively, these data demonstrated that Napabucasin was effective at blocking glioma cell proliferation in vivo.

## Discussion

Although current standard treatments for glioblastoma (GBM, WHO IV) are able to eliminate the majority of tumor cells, recurrence is still inevitable [3]. Many studies have indicated that harboring glioma stem cells (GSCs), a small subpopulation of GBM cells with unlimited self-renewal and the capacity to regenerate tumorigenic progenies, may be a major cause of disease relapse [20]. Therefore, targeting GSCs is a promising and efficacious strategy to eradicate GBM. Signal transducer and activator of transcription 3 (STAT3) is an essential transcription factor that controls many physiological and pathological processes including the cell cycle, cell survival, angiogenesis, and immune responses. Considering the well-documented role of overactive STAT3 signaling in glioma, the therapeutic potential of targeting this pathway should be emphasized. Multiple attempts to develop inhibitors against STAT3 pathway have been reported, and a variety of STAT3 inhibitors, including chemicals, STAT3-binding peptides, and siRNA reagents, have been developed with various degrees of success [21, 22].

Napabucasin (BBI608), a novel STAT3-specific inhibitor, was identified by Li et al. [11]. They revealed that Napabucasin efficiently suppressed metastasis and relapse of a variety of cancers by inhibition of STAT3-driven gene transcription. Importantly, Napabucasin treatment impairs spheroid formation of liver cancer stem cells and downregulates the expression of stemness genes such as SOX2, BMI-1, Nanog, and c-Myc. Considering the promising preclinical data of Napabucasin as both a monotherapy and in combination with conventional chemotherapeutic methods, several clinical trials have been performed [16]. Furthermore, a phase III trial of Napabucasin for refractory colorectal cancer highlighted STAT3 as an essential target for the treatment of patients with elevated pSTAT3 expression [17]. However, the effect of Napabucasin on GBM and GSCs remains unclear.

To our knowledge, this is the first study to evaluate the influence of Napabucasin in the context of GBM. We confirmed that STAT3 is a promoting factor for malignant progression and poor prognosis in glioma patients. We found that treatment of GBM cell lines (U87MG and LN229) with Napabucasin significantly blocked cell proliferation, migration, invasion, and sphere formation. Additionally, Napabucasin arrested the cell cycle and induced apoptosis of GBM cells. Following treatment with Napabucasin, the protein and mRNA expression of STAT3 and stemness-associated genes were obviously decreased. Napabucasin treatment also significantly reduced the expression and phosphorylation of RelA, a key transcription factor of the NF-κB signaling pathway. Moreover, Napabucasin repressed tumor growth in an orthotopic nude mouse model of GBM. One of the advantages of Napabucasin is that it remarkably decreased the protein level of STAT3 and did not simply inhibit the phosphorylation of STAT3. In a study by Zuo et al., the underlying mechanism of the downregulated STAT3 protein level was mediated by protein synthesis inhibition induced by Napabucasin [23]. In our study, we reveal that Napabucasin treatment reduced STAT3 expression at the transcriptional level. Overall, our results support the potential use of Napabucasin as an efficacious anti-glioma therapeutic agent.

## Conclusions

Our results indicate that Napabucasin suppresses the expression of STAT3, and thus impairs the proliferation, migration, and invasion of glioblastoma cells. Napabucasin administration effectively decreases the stemness of glioma cells. Furthermore, we found that Napabucasin significantly impairs the expression and phosphorylation of RelA, which is a novel pharmacological mechanism of Napabucasin. Importantly, treatment with Napabucasin impedes the growth of intracranial U87MG-derived tumors in nude mice. Therefore, Napabucasin may be a promising and potent agent to suppress GBM progression.

## Data Availability

The data supporting the findings of this study is included within the article.

## Abbreviations

EdU: 5-ethynyl-29-deoxyuridine
GBM: Glioblastoma
GSC: Glioma stem cell
IC50: Half maximal inhibitory concentration
OS: Overall survival
STAT3: Signal transducer and activator of transcription 3
TMZ: Temozolomide
